# Economic burden of chronic pulmonary aspergillosis in Uganda: a cost-of-illness analysis

**DOI:** 10.1016/j.ijregi.2026.101007

**Published:** 2026-09-09

**Authors:** Felix Bongomin, Bwambale Jonani, Martha Namusobya, Norman van Rhijn, David W. Denning

**Affiliations:** 1 Department of Medical Microbiology and Immunology, Faculty of Medicine, Gulu University, Gulu, Uganda; 2 Laboratory Department, Sebbi Hospital, Kampala, Uganda; 3 School of Medicine, College of Health Sciences, Makerere University, Kampala, Uganda; 4 Manchester Fungal Infection Group, Division of Evolution, Infection and Genomics, School of Biological Sciences, Faculty of Biology, Medicine and Health, The University of Manchester, Manchester, United Kingdom

**Keywords:** Economic burden, Chronic pulmonary aspergillosis, Catastrophic health expenditure, Productivity losses, Uganda

## Abstract

**Objectives:**

Chronic pulmonary aspergillosis (CPA) is a complication of tuberculosis that is under-recorgnizedin resource-limited settings. We quantified the economic burden of CPA in Uganda from societal, healthcare system, and household perspectives.

**Methods:**

We used a prevalence-based cost-of-illness approach with an estimated 26,765 annual CPA cases, stratified by clinical subtype. Direct medical costs were obtained from local health facilities and Joint Medical Stores pricing. Undiagnosed cases were modeled through a tuberculosis misdiagnosis pathway with 20% annual mortality. Productivity losses were estimated using disability-adjusted life years (DALYs), valued at Uganda’s gross domestic product (GDP) per capita ($1072.70). We used return-on-investment (ROI) analysis to compare full (100%) diagnostic coverage with the current estimated 10%. Structural scenario analyses varied undiagnosed mortality (15-30%), healthcare costs, and baseline coverage.

**Results:**

At 100% coverage, total annual direct medical costs were $20,136,000 and productivity losses were $63,642,000, giving a total economic burden of $83,778,000 (0.16% of GDP), with 1,833 deaths and 59,329 DALYs. At 10% coverage, the burden was $162,749,000 (0.30% of GDP). Expanding coverage from 10% to 100% generated net savings of $78,971,000 (ROI of 4.73:1), robust across all scenarios (range 2.50 - 8.99). Mean out-of-pocket expenditure was $256.47 per patient, representing a catastrophic burden for low-income households. Total burden was most sensitive to annual CPA cases, while mortality among undiagnosed patients most influenced net savings.

**Conclusion:**

CPA imposes a substantial economic burden in Uganda. Underdiagnosis leads to avoidable mortality, productivity losses, and catastrophic costs. Integrating CPA screening into tuberculosis services and expanding diagnostic capacity are justified strategies aligned with Uganda’s universal health coverage goals.

## Highlights

• With 26,765 cases diagnosed and treated, chronic pulmonary aspergillosis (CPA) imposes a $83.78 million annual economic burden in Uganda (0.16% of gross domestic product).
• Productivity losses account for 76.0% of the total CPA burden nationwide.
• Universal diagnosis and treatment would yield net savings of $78.97 million with a 4.73:1 return on investment ratio.
• The poorest income quintile (≤$325 annual income) faces an out-of-pocket expenditure/capacity-to-pay ratio of 138.2%, more than three times the World Health Organization catastrophic threshold of 40%.

## Introduction

Chronic pulmonary aspergillosis (CPA) is a progressive pulmonary disease caused by *Aspergillus* species, characterized by cavities, nodules, or fibrotic changes in the lung parenchyma that persist for at least 3 months [1]. Diagnosis requires microbiological or immunological evidence of *Aspergillus* infection, most commonly elevated *Aspergillus* immunoglobulin G, in the context of pre-existing structural lung disease [1], after exclusion of alternative causes such as active tuberculosis or malignancy.

CPA is estimated to have a global incidence of 1.8 million cases, a prevalence of >6 million cases, and is associated with 8-20% mortality rates [2]. Apart from a small proportion of patients (<5%) who undergo lung resection surgery, the chronic nature of CPA necessitates extended treatment protocols and continuous medical supervision, which amplifies both its clinical significance and economic impact.

The burden of fungal infections is particularly pronounced across sub-Saharan Africa owing to several regional determinants [3]. Among them, the region continues to experience a disproportionate share of the global human immunodeficiency virus (HIV) burden, with approximately 25.6 million affected individuals [4]. Concurrently, tuberculosis incidence rates remain elevated at 206 per 100,000 population across Africa and 197 per 100,000 population in Uganda 5. Previous studies have established that the prevalence of CPA following pulmonary tuberculosis in African populations ranges from 4% to 15%, with significant regional variation [6,7]. Notably, recent estimates have shown an annual prevalence of over 26,765 cases of CPA in Uganda [8].

Evidence from Uganda demonstrates a complex interplay between tuberculosis and subsequent CPA development. Patients with prior tuberculosis (TB) are 6.61 times more likely to develop CPA, with an overall prevalence of CPA among patients with prior TB of 32% [9]. The annual CPA incidence demonstrates disparities, with rates of 6.50% in patients with cavitation versus 0.20% in those without cavitation, highlighting structural lung damage as a critical predisposing factor. Previous findings further suggest that HIV co-infection may potentially modify CPA risk, with a prevalence of 3% in patients with HIV compared with 6.70% in individuals without HIV (*P* = 0.177) [10].

The symptomatic overlap between CPA and tuberculosis presents substantial diagnostic challenges in resource-limited settings [11]. Both conditions manifest as persistent cough, hemoptysis, and weight loss, contributing to the underdiagnosis and undertreatment of CPA, particularly in contexts with limited diagnostic capacity [1,12]. Significant fatigue is prominent in CPA as is major hemoptysis, a highly anxiety-provoking symptom [13]. The potential for clinical confusion between TB and CPA is especially concerning in Uganda, where fungal infection diagnostics remain largely confined to tertiary referral centers despite the country’s high tuberculosis prevalence and consequent CPA risk [14].

Although oral itraconazole, the recommended antifungal therapy for CPA, is available in at least 43% of African countries, its accessibility varies considerably. Treatment costs range from less than $1 in Uganda to $19 in Nigeria for a standard 400 mg daily dose [15]. Nevertheless, the comprehensive economic burden of CPA in Uganda encompassing out-of-pocket expenditures (OOPE), productivity losses, catastrophic health expenditures, and opportunity costs remains undocumented. This knowledge deficit impedes evidence-based policy development and appropriate resource allocation for CPA management within Uganda’s healthcare system.

The present study aimed to estimate the economic burden of CPA in Uganda from societal, healthcare, and household perspectives. This economic analysis will illuminate the potential ROI for an enhanced diagnostic capacity and improved access to affordable antifungal agents within Uganda’s healthcare infrastructure.

## Methods

### Study population and setting

Uganda is a landlocked East African country with an estimated population of 48.2 million according to the 2024 National Population and Housing Census [16]. The predominantly young population (median age 16.7 years) faces a dual burden of communicable and non-communicable diseases. Despite significant progress in health indicators over the past two decades, Uganda continues to contend with a high tuberculosis burden (197 per 100,000 population) [5], which serves as a major risk factor for CPA.

### Modeling CPA cases in Uganda

We used 26,765 annual CPA cases based on a previous risk-deterministic model [8], which accounted for post-TB cases, misdiagnosed TB (19% in patients without HIV and 10% in patients with HIV [12]), dual CPA and TB infections (6.3% in individuals with HIV and 6.20% in individuals without HIV [9]), and TB survivors with an assumed annual incidence rate of 6.5% among those with residual cavities (30% of TB survivors) and 0.2% in those without cavities [10]. The model additionally accounted for CPA cases that occur during or shortly after TB treatment completion (8% [17]). The case estimation was derived primarily from post-TB scenarios and does not explicitly account for CPA secondary to other structural lung conditions.

We stratified these cases by disease subtype using an estimated distribution pattern based on Page et al.'s [10] study from Gulu, Uganda and expert opinion. The distribution used was 16.70% (n = 4470) for simple aspergilloma, 77.20% (n = 20,663) for chronic cavitary/fibrosing CPA, and 6.10% (n = 1632) for cases with azole resistance or contraindication.

### Economic burden analysis framework

We implemented a prevalence-based cost-of-illness methodology from a societal perspective, capturing direct medical costs and indirect costs (productivity losses) within a 1-year analytic horizon. We modeled 11 diagnostic and treatment coverage scenarios from 0% to 100% in 10% increments. The primary comparator for ROI analysis was the current estimated diagnostic and treatment coverage of approximately 10%. All monetary estimates are reported to the nearest $1000.

### Patient classification

At each diagnostic and treatment coverage level, patients were classified into two groups.

Diagnosed and treated patients: those identified through screening and diagnostic services who received guideline-concordant therapy. We mapped typical patient pathways across the three CPA clinical subtypes, identifying healthcare resources utilized at each stage based on the CPA case definition for resource-constrained settings [1], and *clinical guidance for management without mycologist support* [18] (Figure 1).

**Figure 1:**
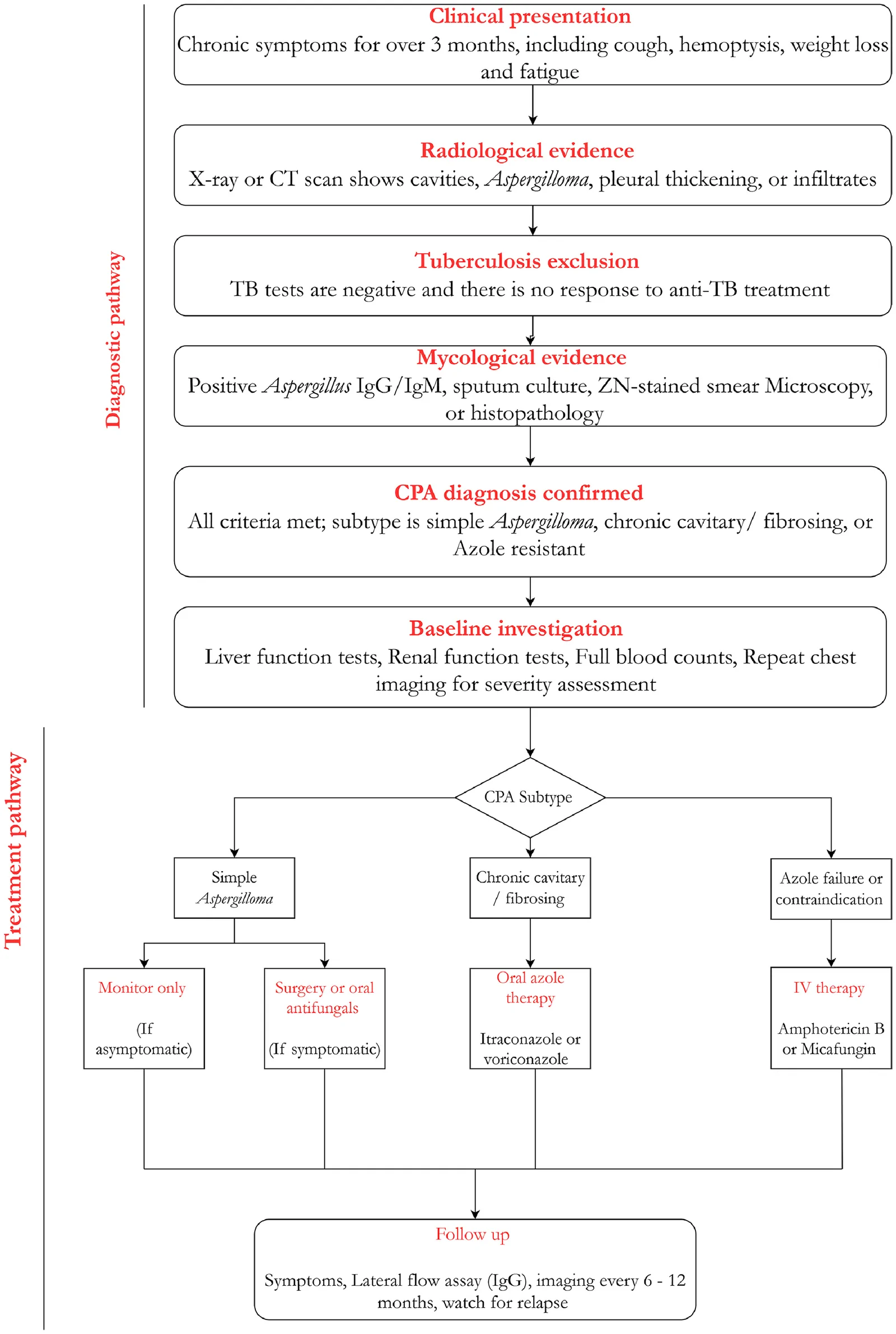
Diagnostic and treatment algorithm for chronic pulmonary aspergillosis in resource-constrained settings. CPA, chronic pulmonary aspergillosis; CT, computed tomography; IgG, immunoglobulin G; IgM, immunoglobulin M; IV, intravenous; TB, tuberculosis; ZN, Ziehl–Neelsen.

Undiagnosed patients: those who remained unidentified due to limited diagnostic capacity were not assumed to have zero healthcare contact. In Uganda’s high-TB-burden context, patients with undiagnosed CPA characteristically present to TB clinics with hemoptysis, cough, and weight loss, receive empiric first-line TB treatment, and undergo chest radiography and sputum microscopy under TB diagnostic protocols. We modeled this misdiagnosis pathway by assigning each undiagnosed patient annual baseline healthcare costs. These included primary-level TB clinic consultations (two visits for simple aspergilloma, three visits for azole failure, and four visits for cavitary/fibrosing CPA), two chest radiographs, two sputum smear microscopy tests, and one course of empiric category I TB drugs at Uganda Joint Medical Stores pricing.

### Direct medical costs

We estimated direct medical costs using approximate prices charged at Ugandan healthcare facilities, comprising diagnostic costs, treatment costs, and follow-up care costs.

For azole-resistant CPA, we modeled diagnostic costs including histopathology for tissue diagnosis. We acknowledge that in practice, these patients would typically have received an initial course of azole therapy before resistance was identified, and our model did not include these prior treatment costs. This represents a limitation and an underestimation of direct costs for this subgroup. We applied a single 10-day course of liposomal amphotericin B (3 mg/kg/day for a 60 kg adult) at Uganda Joint Medical Stores pricing. While we recognize that this may not constitute definitive treatment for all patients, it represents the practical limit of what can be delivered in the Ugandan setting, and some patients may achieve clinical stabilization with this regimen. Repeated courses would add to costs and further strengthen the economic case for earlier diagnosis and susceptibility testing.

### Diagnostic costs

We set outpatient consultation fees at $14 per visit based on local tertiary hospital pricing for specialist consultations. Diagnostic tests were costed using local healthcare market facility charges: chest radiography ($9), computed tomography (CT) scan ($81), TB testing by microscopy ($4.05), *Aspergillus* serology ($8), sputum culture ($14), liver function tests ($14), renal function tests ($14), and histopathology ($27, applied only to azole failure cases requiring tissue diagnosis).

### Treatment costs

Itraconazole was costed at $43 per month using Uganda’s Joint Medical Stores’ pricing. For simple aspergilloma, 90% of patients were assumed to be symptomatic and receive a 6-month itraconazole course, consistent with clinical guidance that recommends antifungal therapy for symptomatic patients. The remaining 10% of patients, who are asymptomatic, are managed with observation alone. This distribution reflects the typical presentation in clinical practice, where most patients with simple aspergilloma seeking care have symptoms such as cough, hemoptysis, weight loss, or fatigue. Surgical resection, while potentially curative for simple aspergilloma, was assigned zero costs as it is not routinely available in Uganda outside a small number of highly specialized tertiary facilities.

For chronic/cavitary CPA, a full 12-month itraconazole course was applied. For cases of azole failure or contraindication (hereafter azole-resistant CPA) , liposomal amphotericin B was considered as the alternative antifungal therapy, dosed at 3 mg/kg/day for a 60 kg adult patient (180 mg/day, approximately 3.6 vials of 50 mg), at a cost of $27.55 per day, totaling $275.50 for a 10-day course at Joint Medical Stores pricing.

### Follow-up care costs

Follow-up care frequency varied by disease subtype reflecting clinical severity. We used two follow-up visits for patients with simple aspergilloma, four visits for patients with azole-resistant CPA (reflecting the need for closer monitoring with amphotericin B therapy and assessment of treatment response), and three visits for patients with cavitary/fibrosing CPA. Follow-up tests included chest X-ray, *Aspergillus* serology, liver function tests (included for all subtypes to reflect monitoring requirements for both itraconazole and amphotericin B), and renal function tests (Supplementary S1 File).

### Productivity losses

For the diagnosed and treated patients, we applied the disability weights (DWs) from the Global Burden of Disease (GBD) study for chronic obstructive pulmonary disease and other chronic respiratory diseases [19]. For simple aspergilloma, we used the DW for mild respiratory diseases (0.019); for azole-resistant CPA, we adopted the DW for moderate respiratory diseases (0.225); and for chronic cavitary/fibrosing CPA we used the DW for severe respiratory diseases (0.408).

DWs for untreated CPA/aspergilloma are not available in the published literature. For this analysis, we extrapolated higher DWs than those for treated patients to reflect progressive symptoms and lack of therapy. Specifically, we applied DWs of 0.05 for untreated simple aspergilloma, 0.400 for untreated azole-resistant CPA, and 0.500 for untreated cavitary/fibrosing CPA. These values were varied by ±20% during sensitivity analyses, to account for uncertainty in their estimation.

### Years of life lost (YLL)

#### Diagnosed patients

We applied subtype-specific mortality rates for diagnosed and treated patients. Published data report 5-year cumulative mortality rates of 11% for simple aspergilloma, 34% for chronic/fibrosing cavitary CPA, and 23% for azole failure/contraindication [20]. The lower mortality for azole-resistant cases reflects clinical heterogeneity within this group, which includes patients with varying degrees of disease severity and some who may have already received initial treatment.

Given our 1-year time horizon, we converted these 5-year mortality rates using exponential survival modeling, assuming constant hazard over time.

$$1\;year\;mortality\;\left(m_{1}\right)=1-\;{\left(1-\;m_{5}\right)}^{1\;/\;5}$$

Where,

$m_{1}\;$= approximate 1-year mortality risk

$m_{5}$ = 5-year cumulative mortality risk

The equation was derived from the exponential survival function $S\left(t\right)=\;e^{-\gamma t}$, with mortality risk m(t)=1−S(t)[21]. The estimated 1-year mortality rates were 2.30% for simple aspergilloma, 7.97% for chronic cavitary/fibrosing CPA, and 5.09% for azole-resistant (as an approximation from sub-acute invasive aspergillosis (SAIA)). For never-diagnosed patients, we applied 20% mortality across all subtypes. We calculated YLL using a median age at death of 41 years [15] and life expectancy in Uganda of 68.7 years, yielding 27.7 YLL per death [22].

#### Undiagnosed patients

We applied a uniform annual mortality rate of 20% across all subtypes. This estimate is conservative relative to the published managed cohort data [23], which reported approximately 14% 1-year mortality in a treated UK cohort. The estimate of 20% accounted for Uganda-specific factors including limited availability of antifungal therapy, late clinical presentation, HIV co-infection, malnutrition, and limited rehabilitation capacity.

#### Years lived with disability (YLD)

We calculated YLD using DWs from the GBD 2015 study [19]. For diagnosed and treated patients, we used weights of 0.019 (simple aspergilloma), 0.408 (chronic/fibrosing cavitary), and 0.225 (azole failure). For never-diagnosed patients experiencing uncontrolled disease progression, we used higher DWs reflecting uncontrolled symptoms, progressive lung destruction, and substantial functional impairment: 0.05 (simple), 0.5 (chronic/fibrosing), and 0.4 (azole failure).

Patients who survived contributed 1 full year to YLD. Patients who died were assumed to have experienced disability for 0.5 years before death, and thus contributed 0.5 years to YLD.

#### Disability-adjusted life years (DALYs)

DALYs were calculated as the sum of YLL due to premature mortality and YLD.

#### Valuation of productivity losses

We valued productivity losses using the human capital approach, multiplying total DALYs by Uganda’s gross domestic product (GDP) per capita ($1072.70 for 2024) [24]. This approach assumed that each DALY represents lost economic productivity equivalent to the per capita economic output.

#### OOPE analysis

To analyze the financial burden of CPA management on households, we assessed OOPE and the prevalence of catastrophic health expenditure using the World Health Organization (WHO) capacity-to-pay approach [25].

#### OOPE

We defined OOPE as direct payments made by individuals to healthcare providers without third-party reimbursement. We utilized the WHO-reported figure indicating that 34.1% of total health expenditure in Uganda is paid out of pocket by patients 26.

OOPE=Directmedicalcosts×0.341

#### Household capacity to pay

We used average annual household income data from the National Labour Force Survey 2021 27. Household capacity to pay was defined as annual household income minus essential food expenditure. Essential food expenditure was estimated as 42.9% of annual household income based on Ministry of Finance and Economic Development data 28). Thus, capacity to pay was calculated as:

$$\begin{array}{ccc} \text{Capacity}\;\text{to}\;\text{pay} & = & \mathrm{Annual}\;\text{household}\;\text{income}\;-\;\text{Food}\;\text{expenditure}\\ & = & \mathrm{Annual}\;\mathrm{household}\;\mathrm{income}\;x\;\left(1-0.429\right) \end{array}$$

#### Catastrophic health expenditure

Following WHO guidelines, we defined the catastrophic threshold as 40% of the capacity to pay [25]. We calculated the OOPE ratio as:

$$OOPE\;Ratio=\;\frac{\text{Out}-\text{of}-\text{pocket}\;\text{expenditure}}{\text{Capacity}\;\text{to}\;\text{pay}\;}\;x\;100\%$$

Households with an OOPE ratio exceeding 40% were classified as experiencing catastrophic health expenditure. We stratified the catastrophic health expenditure analysis by income quintile using quintile-specific income data from the National Labour Force Survey 2021 [27], with annual incomes of $325 for quintile 1 (poorest 20%), $520 for quintile 2, $810 for quintile 3 (middle), $1215 for quintile 4, and $1620 for quintile 5 (richest 20%).

#### Time horizon and discounting

We employed a 1-year time horizon aligned with Uganda's July-to-June fiscal planning cycle, providing actionable data for annual health budgeting processes while capturing the distinct diagnostic and treatment initiation costs of CPA. Given the 1-year timeframe, we did not apply discounting to costs or outcomes.

#### Sensitivity analysis

We conducted one-way sensitivity analyses by varying each parameter across its plausible range while holding all others at base-case values. Parameters affecting the diagnosed patient group were evaluated against the total economic burden at 100% diagnostic and treatment coverage. We varied annual CPA cases by ±25%, subtype-specific diagnosed mortality by ±25%, GDP per capita by ±15%, direct medical costs by ±20%, DWs for treated patients by ±10%, DWs for untreated patients by ±20%, YLL per death by ±10%, and undiagnosed misdiagnosis costs by ±30%. Parameters affecting only the undiagnosed patient group (undiagnosed mortality, DWs for untreated patients, and undiagnosed misdiagnosis costs per patient) were evaluated against net savings from 10% to 100% coverage, rather than absolute burden at 100% coverage, since no undiagnosed patients remain at full coverage. ROI was calculated as net savings (total burden at baseline minus total burden at 100% coverage) divided by the incremental direct costs of moving from 10% to 100% coverage.

In addition, structural scenario analyses varied three core assumptions simultaneously: undiagnosed patient mortality (15%, 20%, 30%), undiagnosed patient healthcare utilization ($0, $48-$62 per patient, $70-$80 per patient) and diagnostic coverage baselines (0%, 10%), to test whether the main outcomes remained robust to different structural framings. All analyses were performed using R version 4.5.2 (R Core Team, R Foundation for Statistical Computing, Vienna, Austria).

#### Model validation

We validated our model structure and assumptions through review by clinical experts on fungal diseases familiar with the Ugandan CPA and TB landscapes (DWD, NVR). We verified mathematical consistency and computational accuracy through independent recalculation of key model components by two authors (BJ, FB).

## Results

### Healthcare context

Uganda operates a decentralized healthcare system structured in tiers, from village health teams at the community level to national referral hospitals. This pyramid structure determines service availability, with specialized diagnostics and treatments primarily concentrated at higher-level facilities. Uganda’s healthcare financing landscape features significant OOPE (34.1% of total health expenditure) [26], while health insurance coverage remains limited to 1.10% of the household population [29], leaving most citizens vulnerable to health-related financial shocks.

The country has expanded its access to TB diagnosis and treatment through a network of 1500 diagnostic and 2200 treatment centers nationwide, with 249 Xpert devices in 227 (15%) TB diagnostic units [30]. In contrast, fungal diagnostic capacity remains severely limited, with conventional fungal cultures available only at the National Referral Hospital and a few regional referral hospitals. *Aspergillus*-specific serological tests have limited availability in private standalone laboratories. CT scanning, which is essential for CPA diagnosis and monitoring, is performed in approximately 25 facilities across the country [31], with a significant concentration in the central region. Treatment options for CPA are similarly constrained, with itraconazole (the first-line oral antifungal) not included in Uganda's Essential Medicines List (EML), despite being on the WHO’s EML since 2017 [32]. Surgical intervention for appropriate cases of simple aspergilloma is only possible at the National Referral Hospital.

### Epidemiological estimates

Based on the previous risk-deterministic model, we estimated 26,765 annual CPA cases in Uganda, distributed as 4470 simple aspergilloma (16.70%), 20,663 chronic cavitary/fibrosing CPA (77.20%), and 1632 azole failure/contraindication (6.10%).

### Direct medical costs

At 100% diagnostic and treatment coverage, total annual direct medical costs of CPA management in Uganda would reach $20,136,000, comprising diagnostic costs ($3,417,000), treatment costs ($12,150,000), and follow-up costs ($4,569,000). Per-patient direct costs varied by clinical subtype: $476 for simple aspergilloma, $665 for azole failure cases, and $819 for chronic/cavitary CPA (Table 1).

**Table 1:** Annual direct medical costs, productivity losses, and total economic burden of CPA in Uganda by clinical subtype and cost category.

Cost item	Simple aspergilloma	Azole failure	Cavitary/fibrosing	Subtotal (all cases)
**A. Direct medical costs**				
**Diagnostic costs**				
Initial consultation	$14	$14	$14	
CT scan	$81	$81	$81	
*Aspergillus* serology	$8	$8	$8	
Sputum culture	$14	$14	$14	
Histopathology	-	$27	-	
Chest X-ray	$9	$9	$9	
**Per-case diagnostic cost**	**$126**	**$153**	**$126**	
**Total diagnostic costs**	**$563,000**	**$250,000**	**$2,604,000**	**$3,417,000**
**Treatment costs**				
Itraconazole - 90% symptomatic (6 months)	$232	-	-	
Itraconazole - 10% asymptomatic	$0	-	-	
Itraconazole - full course (12 months)	-	-	$516	
Liposomal amphotericin B (10-day course)	-	$276	-	
Surgery	$0	$0	$0	
**Per-case treatment cost**	**$232**	**$276**	**$516**	
**Total treatment costs**	**$1,038,000**	**$450,000**	**$10,662,000**	**$12,150,000**
**Follow-up costs**				
Consultations (2 / 4 / 3 visits)	$28	$56	$42	
Chest X-rays	$18	$36	$27	
*Aspergillus* serology	$16	$32	$24	
Liver function tests	$28	$56	$42	
Renal function tests	$28	$56	$42	
Per-case follow-up cost	$118	$236	$177	
Total follow-up costs	$527,000	$385,000	$3,657,000	$4,569,000
**Total direct costs per case**	**$476**	**$665**	**$819**	
**Total direct costs (all cases)**	**$2,128,000**	**$1,085,000**	**$16,923,000**	**$20,136,000**
**B. Productivity losses (diagnosed and treated patients, 100% coverage)**				
Deaths	103	83	1,647	1,833
YLL	2,854	2300	45,638	50,792
Survivors	4,367	1,549	19,016	24,932
Years lived with disability -survivors	83.0	348.5	7,758.5	8,190.0
Years lived with disability - deaths	1.0	9.3	336.0	346.3
Total YLD	84.0	357.9	8,094.5	8,536.3
Total DALYs	2,938	2,658	53,733	59,329
DALYs per case	0.7	1.6	2.6	
**Total productivity loss**	**$3,152,000**	**$2,851,000**	**$**57,639,000	**$**63,642,000
**Productivity loss per case**	**$705**	**$**1,747	**$**2,789	
**C. Total economic burden (100% coverage)**				
Direct costs	$2,128,000	$1,085,000	$16,923,000	$20,136,000 (24.0%)
Productivity losses	$3,152,000	$2,851,000	$57,639,000	$63,642,000 (76.0%)
Total economic burden	$5,280,000	$3,936,000	$74,562,000	$83,778,000

Abbreviations: CPA, chronic pulmonary aspergillosis; CT, computed tomography; DALY, disability-adjusted life year; YLD, years lived with disability; YLL, years of life lost. Values are rounded to the nearest $1,000 thus subtype components may not sum to totals because of rounding.

Undiagnosed patients, modeled through the TB misdiagnosis pathway, incurred annual baseline healthcare costs of $48 per patient for simple aspergilloma, $55 for azole failure cases, and $62 for cavitary/fibrosing CPA. At the current diagnostic coverage, the 90% of patients remaining undiagnosed lead to $1,429,000 in misdiagnosis-related healthcare costs annually.

### Productivity losses

Among diagnosed and treated patients at 100% coverage, an estimated 1,833 deaths would occur annually: 103 from simple aspergilloma, 83 from azole failure cases, and 1,647 from cavitary/fibrosing CPA. This would generate a total of 50,792 YLL. Total DALYs among diagnosed patients amounted to 59,329 (2,938 simple aspergilloma, 2,658 azole failure, 53,733 cavitary/fibrosing CPA), equivalent to productivity losses of $63,642,000. Productivity losses per patient were $705 for simple aspergilloma, $1,747 for azole failure, and $2,789 for cavitary/fibrosing CPA (Supplementary S1 File).

Among undiagnosed patients modeled at 20% annual mortality, an estimated 5,353 deaths would occur annually, 894 among those with simple aspergilloma, 326 among those with azole failure, and 4,133 among cavitary/fibrosing cases. This would lead to 148,332 YLL and 158,419 total DALYs. Total productivity losses attributable to the entirely undiagnosed scenario (0% coverage) amounted to $169,936,000.

### Total economic burden

At the current estimated diagnostic and treatment coverage of 10%, the total annual economic burden of CPA in Uganda is estimated at $162,749,000, equivalent to 0.30% of Uganda’s GDP [33]. It comprises $3,443,000 in direct costs and $159,306,000 in productivity losses. At universal coverage (100%), the total burden falls to $83,778,000 (0.16% of GDP), with direct medical costs rising to $20,136,000 as more patients receive treatment, while productivity losses decline to $63,642,000 as mortality and morbidity are reduced. Productivity losses represent 76.0% of the total burden at 100% diagnostic and treatment coverage.

The relationship between coverage level and each cost component is illustrated in Figure 2. Direct costs, which include both diagnosed patient treatment costs and undiagnosed patient misdiagnosis costs, increase from $3,443,000 at 10% coverage to $20,136,000 at 100%. Productivity losses decline steadily as coverage expands, from $169,936,000 at 0% coverage to $63,642,000 at 100% coverage. Total burden declines steadly as coverage expands, from $171,524,000 at 0% coverage to $83,778,000 at 100% coverage.

**Figure 2:**
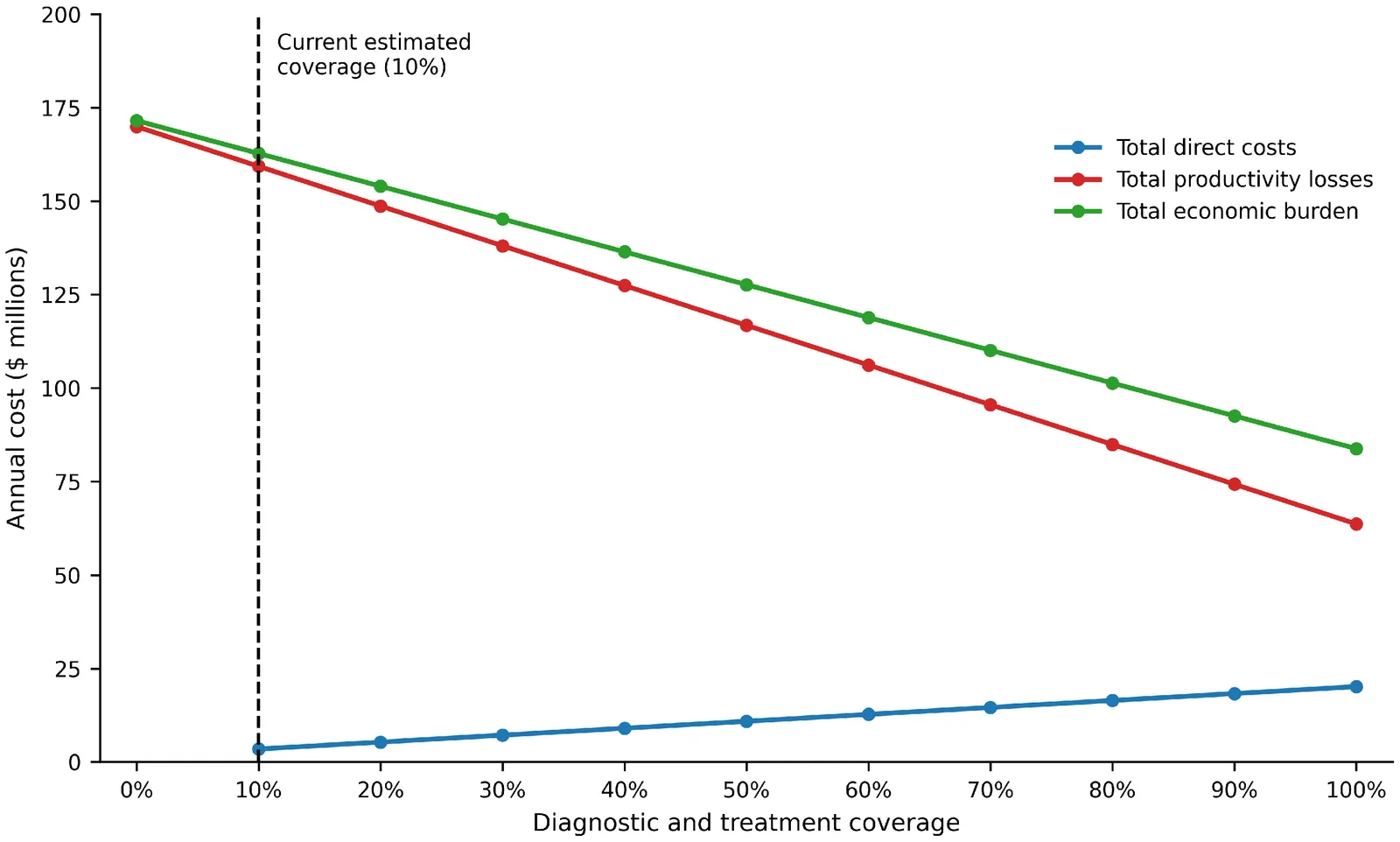
Variation in direct healthcare costs, productivity losses, and total economic burden across diagnostic and treatment coverage rates. The dashed line indicates the current estimated diagnostic coverage in Uganda (10%), which serves as the primary comparator for return-on-investment analysis. Values are rounded to the nearest $1000.

### OOPE

Mean OOPE across all CPA subtypes was $256.47 per patient, varying substantially by clinical presentation: $162.27 for simple aspergilloma, $226.70 for azole failure cases, and $279.20 for chronic/fibrosing CPA. At full diagnosis and treatment of all 26,765 cases, total annual OOPE was estimated at $6,864,000, driven predominantly by chronic cavitary/fibrosing CPA ($5,769,000). The mean OOPE ($256.47) represented 23.9% of Uganda’s GDP per capita per affected individual.

### Catastrophic health expenditure

There were substantial financial inequities across income quintiles. Capacity to pay was $186, $297, $463, $694, and $925 for quintiles 1-5, respectively, after deducting 42.9% of annual household income for essential food expenditure. Using the mean OOPE of $256.47, the corresponding OOPE ratios were 138.2%, 86.4%, 55.5%, 37.0%, and 27.7%, respectively. Quintiles 1-3 exceeded the 40% catastrophic health expenditure threshold, whereas quintiles 4 and 5 remained below the threshold.

### Sensitivity analysis

The total annual economic burden at 100% coverage ranged from $62,811,000 to $104,745,000 around the base case of $83,778,000 (Figure 3a). Annual CPA cases were the most influential parameter (range $41,933,000), followed by diagnosed cavitary/fibrosing CPA mortality (range $24,313,000) and GDP per capita (range $19,093,000).

**Figure 3:**
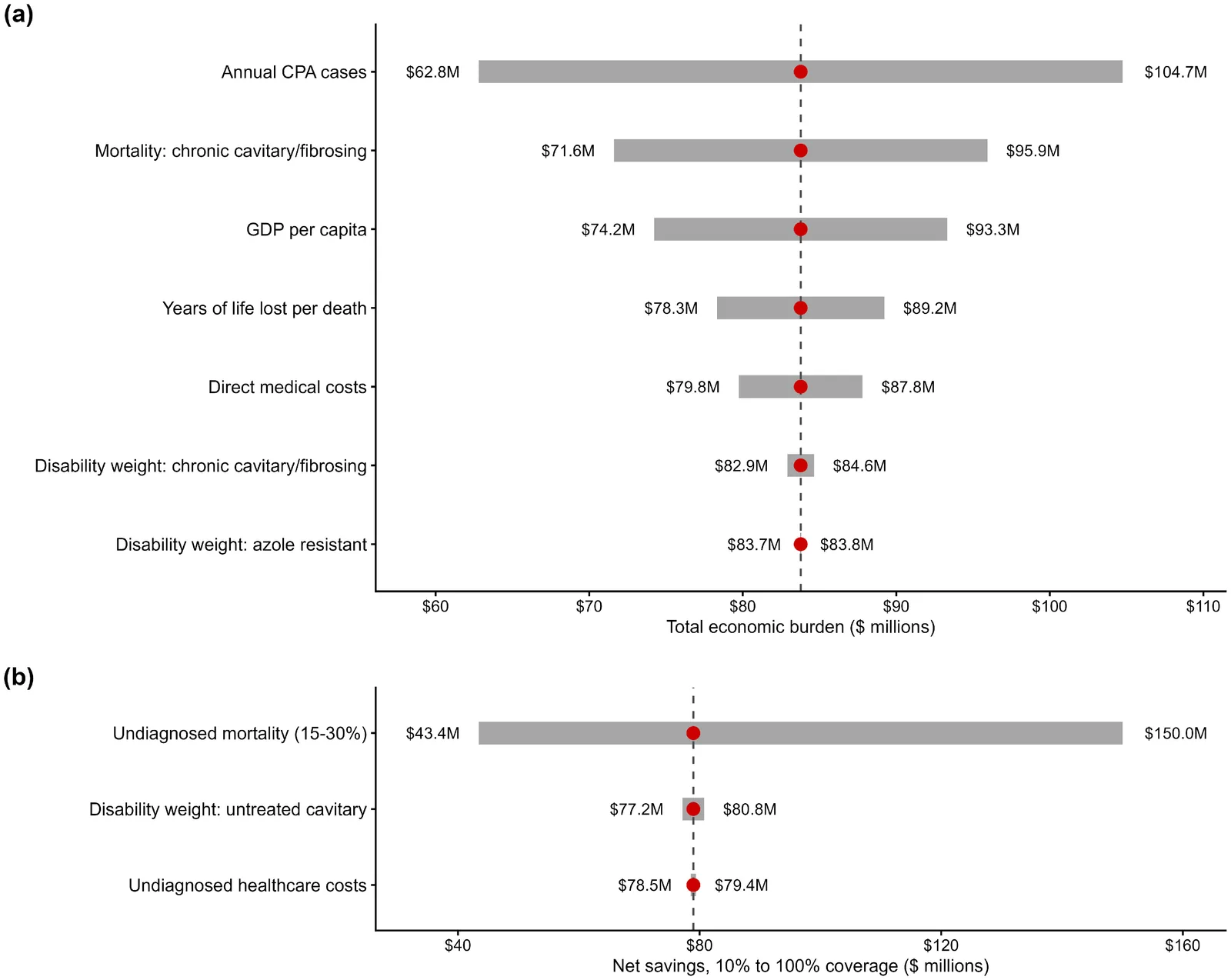
One-way sensitivity analyses for the annual economic burden of CPA in Uganda. (a) Sensitivity at 100% diagnostic and treatment coverage (base case value $83,778,000). (b) Sensitivity of net savings from expanding diagnostic coverage from 10% to 100% (base case $78,971,000). CPA, chronic pulmonary aspergillosis; DW, disability weight; GDP, gross domestic product; M, million.

For net savings from 10% to 100% coverage, undiagnosed patient mortality was the dominant parameter (Figure 3b), with net savings ranging from $43,447,000 to $150,045,000 across the 15-30% range tested. The untreated cavitary disability weight had a modest influence (range $3,591,000), while undiagnosed misdiagnosis costs per patient had minimal effect (range $858,000).

### Structural scenario analysis

Expanding the diagnostic coverage from the current estimated 10% to universal coverage generated positive net savings. The base case model yielded net savings of $78,971,000 and an ROI of 4.73:1. Under optimistic assumptions (15% undiagnosed mortality), net savings were $43,447,000 with an ROI of 2.60:1. Under the pessimistic assumptions (30% undiagnosed mortality rate), net savings rose to $150,045,000 with an ROI of 8.99:1. The conservative scenario combining lower mortality with lower undiagnosed healthcare costs yielded the minimum ROI of 2.50:1 (Table 2).

**Table 2:** Return on investment from expanding CPA diagnostic coverage from 10% to 100%, varying core model assumptions simultaneously.

Scenario	Undiagnosed mortality	Undiagnosed cost/patient	Coverage baseline	Total burden at baseline	Total burden at 100%	Net savings	ROI
Base case	20%	$59/pt	10%	$162,749,000	$83,778,000	$78,971,000	4.73:1
Optimistic: lower mortality	15%	$59/pt	10%	$127,226,000	$83,778,000	$43,447,000	2.60:1
Pessimistic: higher mortality	30%	$59/pt	10%	$233,823,000	$83,778,000	$150,045,000	8.99:1
Conservative: lower mortality + lower costs	15%	$38/pt	10%	$126,725,000	$83,778,000	$42,947,000	2.50:1

ROI (return on investment) = net savings/incremental direct costs from 10% baseline to 100% coverage. Net savings = total burden at baseline − total burden at 100% coverage. All monetary values rounded to the nearest $1000.

Abbreviations: CPA, chronic pulmonary aspergillosis; pt, patient.

## Discussion

This study estimated the economic burden of CPA in Uganda from societal, healthcare system, and household perspectives. Under the current estimated diagnostic coverage of approximately 10%, the total annual economic burden of CPA is $162,749,000, equivalent to 0.30% of Uganda’s GDP. This burden is driven primarily by productivity losses among the large number of undiagnosed patients. Expanding diagnostic and treatment coverage to universal levels would reduce this burden to $83,778,000, generating net savings of $78,971,000 and a ROI of 4.73:1.

The economic rationale for expanding CPA diagnostic capacity is compelling. Every dollar invested in moving from 10% to universal diagnosis generates $5.73 in averted productivity losses. At the individual patient level, diagnosing and treating one patient with CPA costs an average of $752 in direct costs, or $693 net of the $59 in misdiagnosis costs no longer incurred, but prevents $3,971 in lost productivity, a net saving of $3,278 per patient. Structural scenario analyses confirm that this benefit holds across the full plausible range of assumptions, from a conservative scenario of 15% undiagnosed mortality yielding an ROI of 2.50:1, to a pessimistic scenario of 30% undiagnosed mortality yielding 8.99:1. Sensitivity analyses further demonstrated that the ROI benefit was not affected by uncertainty in the cost of misdiagnosing undiagnosed patients.

This ROI is lower than the societal returns of $8 to $54 per health system dollar reported for scaled-up TB screening and preventive treatment in Georgia ($8), Kenya ($27), Brazil ($51), and South Africa ($54) [34]. The estimates are not directly comparable as they were derived from transmission models over a 27-year horizon with discounting at 3% per annum, whereas ours is a one-year, undiscounted estimate that values productivity using the human capital approach. Nevertheless, an ROI of 4.73:1 within a single year is substantial, and the marginal cost of adding CPA diagnosis to existing TB service platforms is low, since patients already attend, chest radiography is already performed, and clinical staff are already engaged. . The primary additional investments in *Aspergillus* serology and CT scanning for complex cases can be integrated without building parallel infrastructure.

Patients with undiagnosed CPA are not absent from the healthcare system. They generate a cost of $1,429,000 annually in misdiagnosis at the current 10% coverage rate through repeated TB clinic attendance, empiric TB treatment and chest radiography that neither resolves their symptoms nor halts disease progression. This reframes the policy question from whether to invest in CPA diagnosis to how to redirect resources already being consumed by misdiagnosis toward an intervention that is both clinically effective and economically efficient.

Productivity losses account for 76.0% of the total economic burden at 100% diagnostic and treatment coverage, and 97.9% at the current coverage (10%) where undiagnosed mortality drives additional YLL. This pattern, in which indirect costs exceed direct medical costs, is consistent with cost-of-illness studies of other chronic infectious diseases in sub-Saharan Africa, where premature mortality in working-age populations generates large productivity losses relative to the modest cost of treatment [35], [36]. The median age at CPA diagnosis of 41 years means that deaths occur at peak productive age, each generating 27.7 YLL, with cavitary/fibrosing CPA contributing90.6% of the total diagnosed productivity losses given its dominant case volume. Scaling up CPA diagnosis would therefore not be primarily about reducing treatment costs or hospital admissions, but preventing deaths in working-age adults and preserving the productive capacity of households already bearing a disproportionate HIV and TB burden [37,38].

Mean OOPE of $256.47 per patient, rising to $279.20 for cavitary/fibrosing CPA, reveals a severe financial protection failure. Under Uganda’s current healthcare financing structure where 34.1% of the total health expenditure is paid out of pocket [26], and only 1.1% of the population has health insurance [29], CPA treatment imposes a catastrophic financial burden on the lowest three income quintiles [27], leaving the majority of Uganda’s population unprotected against CPA costs. This aligns with the broader patterns across sub-Saharan Africa, where out-of-pocket health expenditure remains the primary driver of poverty [35], and where financial catastrophe falls disproportionally on households already at highest biological risk (those with prior tuberculosis and consequent structural lung damage).

We note that itraconazole is not currently included on Uganda’s EML despite WHO listing it since 2017 [32]. Inclusion would enable bulk procurement and generic substitution, reducing the 12-month treatment cost from $516 to approximately $240 per patient. Combined with the expansion of digital chest radiography that has artificial intelligence-assisted interpretation, and selective use of CT scanning for complex cases, the total per-patient costs for cavitary/fibrosing CPA would fall from $819 to approximately $480, bringing OOPE below the catastrophic threshold for middle-income households. A fully subsidized program targeting the lowest two income quintiles [27] would cost approximately $8,150,000 annually, an investment that is programmatically feasible within Uganda’s existing TB and HIV service platforms, which already operate 1500 diagnostic and 2200 treatment centers [30]. Second-line treatments for azole-resistant cases remain challenging as liposomal amphotericin B requires inpatient administration and close monitoring. Comparative cost-effectiveness analyses of second-line options in resource-limited settings are needed to guide procurement decisions for this subgroup.

Therefore, integration of CPA screening and diagnosis into Uganda’s existing TB diagnostic infrastructure is an immediate policy priority. Patients with prior TB are 6.61 times more likely to develop CPA [9], and the symptomatic overlap between the two conditions is a primary driver of persistent underdiagnosis. Integrating *Aspergillus* serology at the TB clinic level, reserving CT scanning for diagnostic uncertainty, and ensuring itraconazole availability through a national essential medicines framework would address the three main access barriers simultaneously.

Our model does not assume that treated patients with CPA return to full health or pre-morbid productivity. Rather, we applied GBD-derived DWs that reflect ongoing functional impairment even with treatment (0.019 for simple aspergilloma, 0.225 for azole-resistant CPA, and 0.408 for cavitary/fibrosing CPA). These weights are substantially lower than those applied to untreated patients (0.050, 0.400, and 0.500, respectively), capturing the partial but meaningful benefit of antifungal therapy. The productivity gains from expanding diagnostic coverage are driven primarily by reductions in premature mortality (YLL accounting for 86% of total DALYs), as treated patients experience lower mortality rates than undiagnosed patients who continue to progress.

Our 1-year time horizon, while aligned with Uganda's fiscal planning cycle, does not capture the cumulative multi-year costs of long-term CPA treatment. For patients requiring therapy beyond 12 months, direct treatment costs would be higher than our estimates. However, this should not be interpreted as undermining the economic case for investment. Firstly, not all patients require lifelong treatment; patients with simple aspergilloma recieve a 6-month course and may discontinue therapy after symptom resolution, while patients with cavitary/fibrosing CPA who stabilize may require less intensive monitoring over time. Secondly, the mortality and morbidity benefits of treatment extend beyond the first year, generating additional productivity gains that would further improve the ROI. Our 1-year estimates therefore provide a conservative assessment of the total benefit of expanding diagnostic coverage.

Our modeling of azole-resistant CPA assigned a treated disability weight of 0.225, reflecting the clinical heterogeneity of this subgroup, which includes patients with varying degrees of disease severity. The cost estimates for this subgroup are conservative, as we did not include the prior azole therapy that would typically have been administered before resistance was confirmed. Including these costs would increase the estimated direct medical burden. We also acknowledge that a single 10-day course of liposomal amphotericin B may not be sufficient for definitive treatment in all cases, and repeated courses would further increase costs, highlighting the need for improved diagnostic capacity and access to alternative antifungal agents.

### Limitations

The 26,765 annual CPA case estimate is derived from a model based on post-TB scenarios and may underestimate the true burden by not accounting for CPA secondary to chronic obstructive pulmonary disease, bronchiectasis, or other structural lung diseases unrelated to TB. The annual CPA case estimate was the most influential parameter in sensitivity analyses, which makes improved national surveillance integrated into TB programs even more necessary. The 20% annual mortality rate applied to undiagnosed patients remains an estimate in the absence of published data on untreated CPA mortality in resource-limited settings, yet it was the most influential parameter on net savings estimates. DWs for untreated CPA are unavailable in published literature and were extrapolated through clinical expert consensus. However, their influence on net saving was modest relative to the mortality assumption. The 1-year time horizon does not capture cumulative multi-year disease costs, long-term disability consequences among survivors, or the capital costs of building diagnostic infrastructure. Surgery costs were set to zero, reflecting the practical unavailability of pulmonary resection in the majority of Ugandan facilities, an assumption that would not hold in settings with greater surgical capacity. Caregiver time, psychological burden, and transportation costs were not captured, and productivity losses among caregivers were excluded, which may imply a higher than estimated true burden. Household capacity to pay was calculated by applying a single average food expenditure share (42.9% of income) to all income quintiles, and the mean OOPE was applied uniformly. In practice, food shares are likely to be higher in lower-income households and lower in higher-income households, so capacity to pay may be overestimated for the poorest quintiles and underestimated for the richest, and the quintile-specific OOPE ratios should be interpreted as approximate. Generalizability to other resource-limited settings with different TB burdens requires local adaptation of cost inputs and healthcare system structures.

### Future directions

Several priority research directions emerge from this analysis. Prospective cohort studies documenting the natural history of untreated CPA in African populations would provide more accurate mortality and morbidity estimates. Such studies face obvious ethical constraints but might be feasible through retrospective focus on patients who are diagnosed late in their disease course or those who die shortly after diagnosis, providing insights into the consequences of delayed diagnosis. Additionally, retrospective case series examining patients initially misdiagnosed as having treatment-resistant tuberculosis but later confirmed to have CPA could illuminate the clinical trajectory and outcomes when appropriate antifungal therapy is delayed. Implementation studies examining optimal strategies for integrating CPA screening into existing TB programs deserve attention. Given the concentration of diagnostic capacity at TB treatment sites within the country and the high-risk populations they serve, TB programs represent a logical platform for CPA case-finding. Cost-effectiveness analyses comparing different screening algorithms, diagnostic technologies, and service delivery models would inform efficient scale-up strategies.

Studies evaluating the acceptability and feasibility of point-of-care diagnostic tools for CPA in resource-limited settings are needed. While aspergillosis serology remains the cornerstone of CPA diagnosis, its requirement for laboratory infrastructure and cost limit accessibility. Lateral flow assays or other simplified diagnostic kits suitable for lower-level facilities could facilitate the expansion of testing services.

## Conclusion

CPA imposes a substantial economic burden on Uganda, estimated at $162,749,000 annually at the current diagnostic and treatment coverage of 10%. This is equivalent to 0.30% of Uganda’s GDP with productivity losses accounting for 97.9% of the burden. The inverse relationship between diagnostic coverage and total economic burden demonstrates that underdiagnosis is not only a clinical failure but a measurable economic inefficiency with consequences for households, the healthcare system, and national productivity. Integrating CPA screening and diagnosis into existing TB diagnostic services, adding itraconazole to Uganda’s EML to enable affordable access, and expanding *Aspergillus* serology capacity at TB clinics serving high-risk post-TB populations would simultaneously address the three barriers to CPA diagnosis and treatment.

## Funding

This research did not receive any specific grant from funding agencies in the public, commercial, or not-for-profit sectors.

## Ethical approval and consent to participate

Not applicable.

## Consent for publication

Not applicable.

## Availability of data and materials

All data generated or analyzed during this study are included in this published article.

## CRediT authorship contribution statement

**Felix Bongomin:** Conceptualization, Data curation, Formal analysis, Funding acquisition, Investigation, Methodology, Project administration, Resources, Software, Supervision, Validation, Visualization, Writing – original draft, Writing – review & editing. **Bwambale Jonani:** Conceptualization, Data curation, Formal analysis, Funding acquisition, Investigation, Methodology, Project administration, Resources, Software, Supervision, Validation, Visualization, Writing – original draft, Writing – review & editing. **Martha Namusobya:** Investigation, Methodology, Writing – original draft, Writing – review & editing. **Norman van Rhijn:** Conceptualization, Data curation, Formal analysis, Funding acquisition, Investigation, Methodology, Project administration, Resources, Software, Supervision, Validation, Visualization, Writing – original draft, Writing – review & editing. **David W. Denning:** Conceptualization, Data curation, Formal analysis, Funding acquisition, Investigation, Methodology, Project administration, Resources, Software, Supervision, Validation, Visualization, Writing – original draft, Writing – review & editing.

## Declaration of competing interest

The authors have no competing interests to declare.

## Appendix Supplementary materials

Supplementary S1 File. Cost data and clinical parameters used in the economic burden analysis of chronic pulmonary aspergillosis in Uganda**.** Image, application 1
